# Neutronic investigation of alternative & composite burnable poisons for the soluble-boron-free and long life civil marine small modular reactor cores

**DOI:** 10.1038/s41598-019-55823-2

**Published:** 2019-12-20

**Authors:** Syed Bahauddin Alam, Bader Almutairi, Tuhfatur Ridwan, Dinesh Kumar, Cameron S. Goodwin, Kirk D. Atkinson, Geoffrey T. Parks

**Affiliations:** 1 Department of Engineering, University of Cambridge, Cambridge, CB2 1PZ United Kingdom; 2 Department of Physics and Astronomy, Uppsala University, Uppsala, Sweden; 3 Department of Nuclear Engineering, Missouri S&T, Missouri, USA; 4 University of Ontario Institute of Technology, 2000 Simcoe Street North, Oshawa, Ontario L1G 0C5 Canada; 5 Rhode Island Nuclear Science Centre, 16 Reactor Road, Narragansett, RI 02882 USA

**Keywords:** Nuclear fuel, Nuclear fusion and fission

## Abstract

Concerns about the effects of global warming provide a strong case to consider how best nuclear power could be applied to marine propulsion. Currently, there are persistent efforts worldwide to combat global warming, and that also includes the commercial freight shipping sector. In an effort to decarbonize the marine sector, there are growing interests in replacing the contemporary, traditional propulsion systems with nuclear propulsion systems. The latter system allows freight ships to have longer intervals before refueling; subsequently, lower fuel costs, and minimal carbon emissions. Nonetheless, nuclear propulsion systems have remained largely confined to military vessels. It is highly desirable that a civil marine core not use soluble boron for reactivity control, but it is then a challenge to achieve an adequate shutdown margin throughout the core life while maintaining reactivity control and acceptable power distributions in the core. High-thickness ZrB_2_ 150 μm Integral Fuel Burnable Absorber (IFBA) is an excellent burnable poison (BP) candidate for long life soluble-boron-free core. However, in this study, we want to minimize the use of 150 μm IFBA since B-10 undergoes an (n, α) capture reaction, and the resulting helium raises the pressure within the plenum and in the cladding. Therefore, we have considered several alternative and novel burnable BP design strategies to minimize the use of IFBA for reactivity control in this study: (Case 1) a composite BP: gadolinia (Gd_2_O_3_) or erbia (Er_2_O_3_) with 150 μm thickness ZrB_2_ IFBA; (Case 2) Pu-240 or Am-241 mixed homogeneously with the fuel; and (Case 3) another composite BP: Pu-240 or Am-241 with 150 μm thickness ZrB_2_ IFBA. The results are compared against those for a high-thickness 150 μm 25 IFBA pins design from a previous study. The high-thickness 150 μm 25 IFBA pins design is termed the “IFBA-only” BP design throughout this study. We arrive at a design using 15% U-235 fuel loaded into 13 × 13 assemblies with Case 3 BPs (IFBA+Pu-240 or IFBA+Am-241) for reactivity control while reducing 20% IFBA use. This design exhibits lower assembly reactivity swing and minimal burnup penalty due to the self-shielding effect. Case 3 provides ~10% more initial (beginning-of-life) reactivity suppression with ~70% less reactivity swing compared to the IFBA-only design for UO_2_ fuel while achieving almost the same core lifetime. Finally, optimized Case 3 assemblies were loaded in 3D nodal diffusion and reactor model code. The results obtained from the 3D reactor model confirmed that the designed core with the proposed Case 3 BPs can achieve the target lifetime of 15 years while contributing to ~10% higher BOL reactivity suppression, ~70% lower reactivity swings, ~30% lower radial form factor and ~28% lower total peaking factor compared to the IFBA-only core.

## Introduction

In recent decades, there have been excessive carbon dioxide emissions due to the ever-growing global population. This growth comes naturally with an increasing demand for energy. Most of the energy produced these days come from hydrocarbon-based fuels that contribute devastatingly to climate change. With the world economy’s flourishing, international trading between countries has been growing progressively as well. International trading has been identified as one of the leading factors that are driving the world’s economy within which seaborne trade takes a large share. At present, 99% of the total freight commercial shipping is driven by low to medium speed diesel engines in which they are responsible for a total of 3% of worldwide carbon dioxide emission^1,2^. Though alternative fuels are being considered including solar, biofuels and wind, none of these is particularly promising in the context of 80% reduction in carbon emission by 2050, which is the target for many developed countries. Nuclear power has the ability to fill this gap in energy technology^1,2^. Nuclear power contributes no CO_2_ emission; henceforth, nuclear energy is recognized as clean energy. Nuclear power has been into existence for peaceful applications for over 60 years. Apart from nuclear power stations, nuclear power is also used for shipping propulsion–while mainly for military purposes. Nuclear propulsion research that was launched by the United States in the early 1940s motivated the development of Pressurized Water Reactor (PWR)^1,3,4^. In the early 50s, the development of naval nuclear propulsion resulted in the lunch of the USS Nautilus in 1955. Since then, nuclear-powered ships had been without any major accidents. This fact demonstrates that nuclear, marine propulsion is a reliable-safe option^1,5^.

There are about 140 nuclear-powered ships (12,000 reactor years of experience) with the vast majority of these being military vessels: submarines, aircraft carriers or cruisers^2^. Few civil or commercial ships have been equipped with nuclear propulsion. Most importantly, apart from the significant environmental benefits, nuclear propulsion exhibits marked stability in the face of volatile fuel prices because of its unique price structure. According to the US Nuclear Energy Institute, nuclear fuel only accounts for ~14% of the total costs of nuclear energy^6^; as a result, changes in the price of primary resources have little effect on operating costs. In contrast, fossil fuel prices have historically experienced dramatic fluctuations: for instance, the price of heavy fuel oil nearly tripled between January 2007 and July 2008^6,7^. It is, therefore, a detailed design of advanced nuclear technology that is cost-effective will result in a superior nuclear propulsion system than the current utilized conventional propulsion systems^6,7^. The key economic advantage of a nuclear-powered container ship is that it can practically double its yearly revenue compared to a conventional fossil-fueled vessel. Besides, its high-speed and flexible operation may allow ships to compete with aircraft in the market of international deliveries. Nonetheless, nuclear power has not been widely applied to shipping for several key reasons: (1) Almost all the experience with marine reactors has been with highly enriched uranium. Civil shipping reactors will require a low enriched core design, which also has a long operating lifetime; (2) The need for an international infrastructure to operate, maintain and support such reactors with its attendant cost; (3) Nuclear licensing and regulation has national variations. Harmonisation of nuclear regulations or the mutual recognition of different licensing standards would be required for significant application of nuclear power to shipping. The solutions of the aforementioned technical issues are hindered by the demands of the aboard environment that results in the following: the limitation of space and weight; rolling and pitching issues; and safety and shielding concerns^8^. These challenges can be potentially solved by a promising design of PWR–small modular reactors (SMR)^9–16^.

One of the main reactor core design challenges is to develop an efficient reactivity control system^9,10^. Generally, traditional PWRs use a combination of three methods of reactivity control: moveable control rods, burnable neutron absorbers, and soluble neutron absorbers. PWRs use soluble boron for uniform power suppression throughout the core by suppressing excess reactivity. There are, however, several benefits for the elimination of soluble boron from reactors such as improved safety; simplification of the overall reactor design by reducing the purification systems with their related components; space reduction; and elimination of the degradation effects of soluble boron on the reactor components. A fundamental strategy of designing a reactor core to be soluble-boron-free (SBF) is the increasing dependence on control rods and burnable poisons (BPs) which both are local neutron absorbers. During regular operation, control rods are inserted in a calculated manner in order to control the reactor power; however, their presence distorts the axial power profile negatively. This, in turn, results in undesirable power peaks and consequently, there is a reduced operating margin which ultimately leads to a significant economic loss. The main challenge in designing any SBF reactor is, therefore, to minimize power peaks while maintaining a proper axial power profile. Besides, relying strongly on moveable control rods would add complexity to the reactor’s control system. It will also elevate the risk of having rod-associated accidents. Hence, BPs perform a significant role in the reactivity control scheme of our SBF civil marine core design.

This study, therefore, focuses on the design of 333 MWth^9,10^ SBF and long life civil marine SMR core while utilizing LEU. We set a target operational life of at least 15 years under high irradiation of around 100 GWd/tonne and constrain uranium enrichment at 20%– in line with proliferation resistance related limits on low-enriched uranium (LEU)^9–12^. It is noteworthy to mention that reactor cores for the purpose of civil marine applications cannot be conceivably as highly enriched as the reactor cores used in military vessels^9,10,17^. Civil shipping reactors will require a LEU core design but should also have a long operating lifetime. Higher fissile loading of 15% U-235 enriched UO_2_ fuel is used in this study to achieve the minimum core life of 15 years^18^. In this study, we investigate BP designs specifically for our SBF SMR core evaluating their reactor physics performance according to the following factors: beginning-of-life (BOL) reactivity suppression, reactivity swing, cycle length, and power peaking factor (PPF). In the previous studies^9,18,19^, we chose high-thickness 150 μm 25 IFBA pins as our BP design. The high-thickness 150 μm 25 IFBA pins design is termed the “IFBA-only” BP design throughout this paper. This paper considers boron 95% enriched in B-10 throughout our IFBA designs for increasing the effectiveness of neutron capture. The main objective of this paper is to minimize the use of 150 μm IFBA since B-10 undergoes an (n, α) capture reaction, and the resulting helium raises the pressure within the plenum and in the cladding. Therefore, in order to minimize the numbers of IFBA-only pins while maintaining an acceptable power profile, this paper presents an investigation of several novel types and strategies of BP loading scheme. These are: (Case 1) a composite BP: gadolinia (Gd_2_O_3_) or erbia (Er_2_O_3_) with 150 μm thickness ZrB_2_ IFBA; (Case 2) Pu-240 or Am-241 mixed homogeneously with the fuel; (Case 3) another composite BP: Pu-240 or Am-241 with 150 μm thickness ZrB_2_ IFBA. All the BP candidates are compared with the reference high-thickness IFBA-only (150 μm 25 IFBA pins) BP design for UO_2_ fuel. Finally, we observe the neutronic performances of the best performing BP candidate at the whole-core level.

The scope of this current study is only limited to the neutronic feasibility of the proposed alternative and novel candidate BPs for the proposed civil marine SBF, SMR core. It is important noting that this paper has not considered the neutronic and practical implications (availability of the fuel, problems of separation and manufacture) and proliferation concerns of these proposed BPs.

## Design and Calculational Methods

In this study, we have used deterministic reactor physics code WIMS 10^20,21^ for the lattice-level calculation while considering JEF 2.2 nuclear data library and 6 energy group structure^9–12,17,22–24^. WIMS performs deterministic neutron transport calculations for every pin in a fuel assembly using an established calculation route through sequence of separate modules^19,20,22,25,26^. However, it does these calculations for an infinitely large core, neglecting the finite height and radius of an actual reactor. Hence, WIMS cannot describe effects such as neutron leakage or power peaking. To explore these essential aspects of reactor design, we use the whole-core code PANTHER (PWR and AGR Neutronics and Thermal Hydraulics Evaluation Route)^27^, which is a FORTRAN-based advanced 3D nodal code. PANTHER approximates each subassembly in a core as a single simple entity with certain neutronic properties. WIMS module “LED” prepares data and allows to load WIMS assemblies into a PANTHER core. To specify PANTHER input, there are four general steps^6,19,28,29^, which need to be repeated for every different subassembly design:

1. To specify the subassembly design in WIMS.
2. To modify the WIMS output with physical data from the assembly (such as fuel mass, density, etc) to create a physical and neutronic description of the reactor that can be read in PANTHER.
3. To develop a thermal-hydraulic model for the assembly which specifies the power density of the fuel at different radii in the pellet, and the thermal conductivity and specific heat capacity for the specific fuel composition.
4. To specify core loading and thermal-hydraulic parameters. These include the layout of the assemblies in the core, the locations of each type of assembly the thermal-hydraulic model for each type of assembly, the location of control rods, and core thermal-hydraulic data.

**Figure 1:**
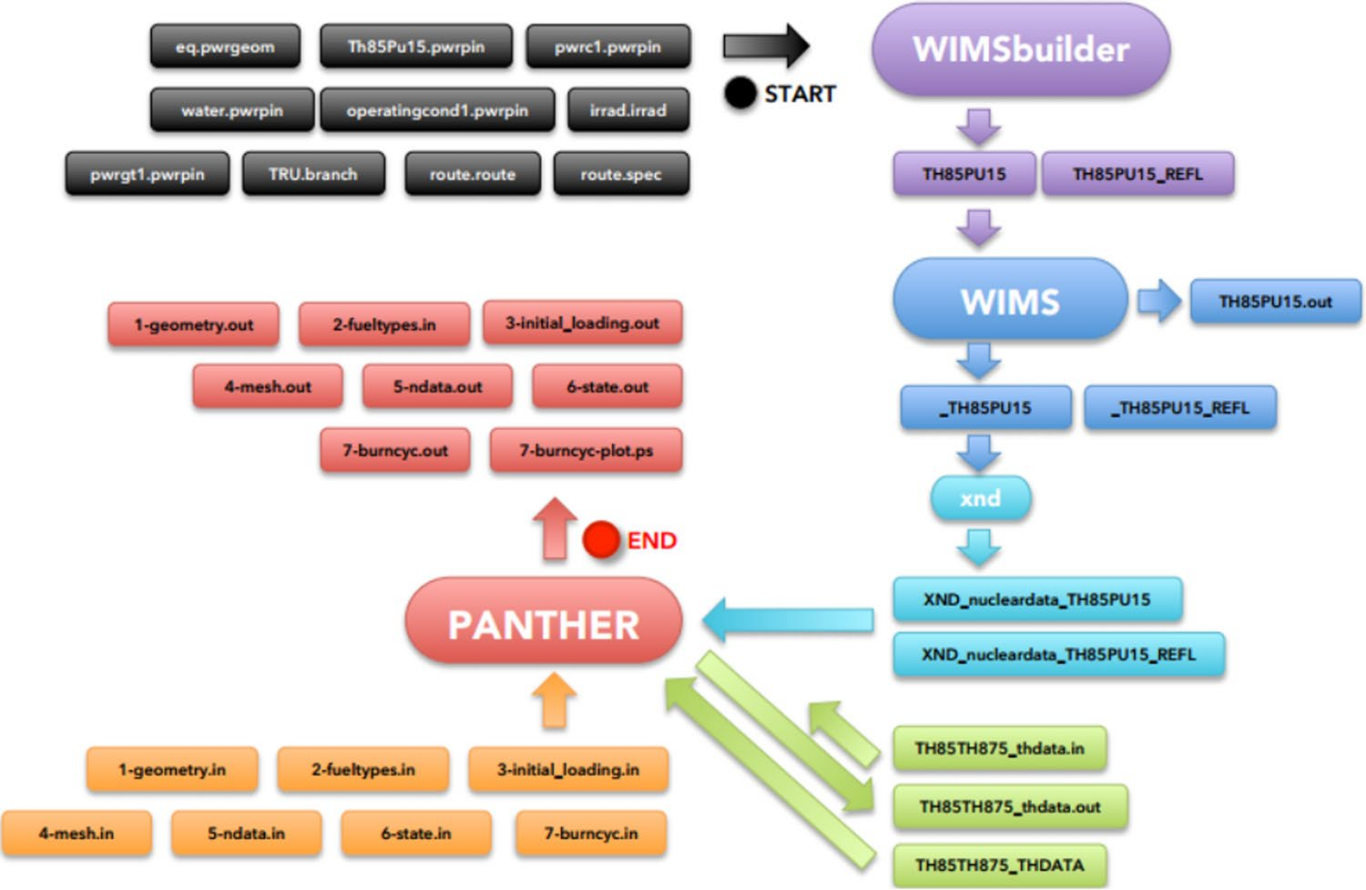
Interconnectivity of the 2D lattice code WIMS and 3D whole-core code PANTHER for the proposed SMR core design^42^.

Figure 1 shows the interconnectivity of the 2D lattice code WIMS and 3D whole-core code PANTHER.

For the analyses of alternative burnable poison, this study considers 15% U-235 enriched homogeneously mixed UO_2_ fuel enriched in in a 13 × 13 assembly while assuming 7.5% neutron leakage and discharge burnup limit of 100 GWd/tonne in the WIMS model^22^. The detailed computational and design methods are reported in our previous studies^9–12,17,22,30^. Table 1 shows the 2D lattice-level (WIMS) and 3D whole-core (PANTHER) system parameter values^12,19,30–38^. It is also important addressing that the results of this article have been introduced in the PhD thesis^19^ of the corresponding author and also in a conference paper^34^.

**Table 1:** 3D whole-core (PANTHER) and 2D lattice-level (WIMS) system parameter values^9–12^.

3D whole-core (PANTHER) parameters		2D lattice-level (WIMS) parameters	
Parameters	Values	Parameters	Values
Thermal power (MWth)	333	Assembly geometry	13 × 13
Coolant pressure (MPa)	155	Control rods per subassembly	16
Soluble boron concentration (ppm)	0	Pin pitch (cm)	1.265
Total coolant flow rate (kg/s)	8370	Pellet diameter (cm)	0.819
Core inlet temperature (°C)	295	Cladding thickness (cm)	0.0605
Number of reactor channels	164	Cladding gap thickness (cm)	0.00497
Number of core channels	112	Total rod diameter (cm)	0.95
Number of channel columns	14	Pitch/diameter (P/D) ratio	1.33
Number of channel rows	14	Hydrogen-to-heavy metal (H/HM) ratio	4
Active core height (m)	1.790	Assembly side length (m)	0.1645
Core diameter (m)	1.970	Assembly area (cm^2^)	300

## Alterative and Composite Burnable Poison Candidates

Our previous studies^9–12,18,19^ demonstrated that using localized, self-shielded gadolinia is an effective means of moderate, long-term reactivity control. However, it has a number of drawbacks. Inherently, it reduces the thermal conductivity of the fuel, and it displaces fissile material, leading to reduced critical lifetime. Additionally, the heavy poison loadings required for long-life cores also lead to severe power peaking within the assembly. To avoid these shortcomings, we explored the feasibility of applying a layer of neutron poison to the outer surface of the fuel pellet in our previous studies^9–12,18,19^. The most prominent industrial implementation of this strategy is Westinghouse’s IFBA. In a typical IFBA assembly, a very thin adhesive coating (1 mg/cm of pellet length ~590 nm thickness) of ZrB_2_ is sprayed onto the outer surface of a normal pellet. This offers important benefits over traditional BP: 1. The poison is completely depleted, and hence there are no residual effects on reactivity at end of life (EOL); 2. ZrB_2_ is coated on the outside of an otherwise normal pin, so there is no change in fuel conductivity, no displacement of fissile material, and no need for separate manufacturing/handling of poison pins. In our SBF marine core^18^, it required 25 absorber pins with 150 μm IFBA to suppress the initial reactivity and the assembly power peaking at 1.29 is 18% higher than for Westinghouse civil IFBA assemblies (assembly PPF = 1.10). Unfortunately, in this thick-coating example, a higher PPF is seen due to the very strong neutron absorption effect. Therefore, in this study minimizing the core PPF will be an important goal. Furthermore, we have observed that “gray” absorbers (erbia) are best suited to trimming excess reactivity early in life (since they do not burn quickly) and “black absorbers” (gadolinia) are best used later in life (because of their lower burnup penalty). In this study, a composite BP is designed to take advantage of both erbia and IFBA, where the erbia provides extended reactivity control and the IFBA provides bulk reactivity suppression and longer cycle length. In addition, we will consider IFBA with gadolinia in order to observe the effect of a black absorber with the high coating BP. As addressed, in this study, all the IFBAs are high-thickness 150 μm IFBA.

**Figure 2:**
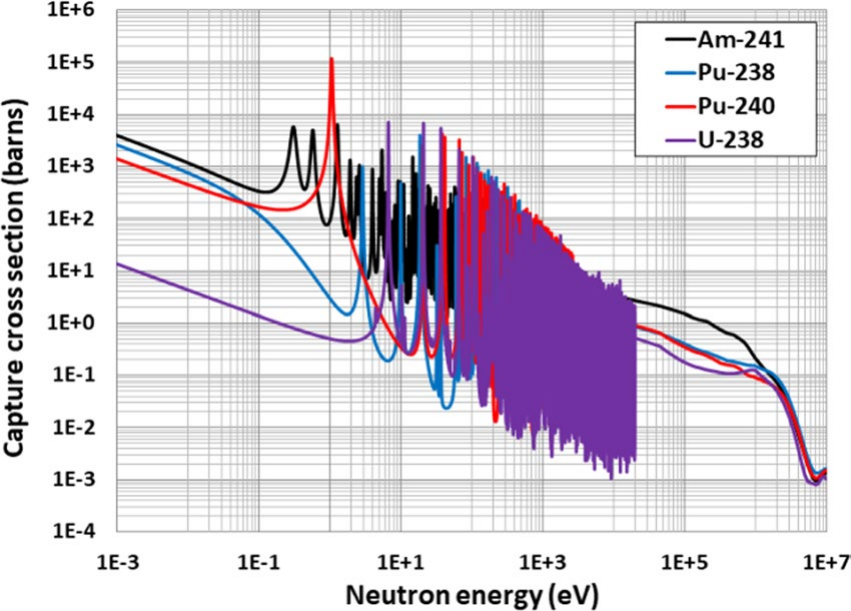
Neutron capture cross sections (barns) of candidate burnable absorber nuclides.

In order to suppress excess reactivity and reduce the through-life reactivity swing, this BP study also pursues the novel technique of mixing Pu-240 homogeneously to the UO_2_ fuel^39^. The BOL reactivity can be suppressed significantly by the addition of Pu-240 to the fuel. This is due to the fact that Pu-240 has significantly higher neutron capture cross section than that of the U-238 in the thermal energy range, as shown in Fig. 2. We will also investigate Am-241, which has capture cross section of ~800 barns at ~0.02 eV (Fig. 2) while the generation of Am-242m has excellent fissile properties^40^. Therefore, it is evident that Am-241 can be a potential candidate to serve effectively as a BP for our SBF core^40^.

**Figure 3:**
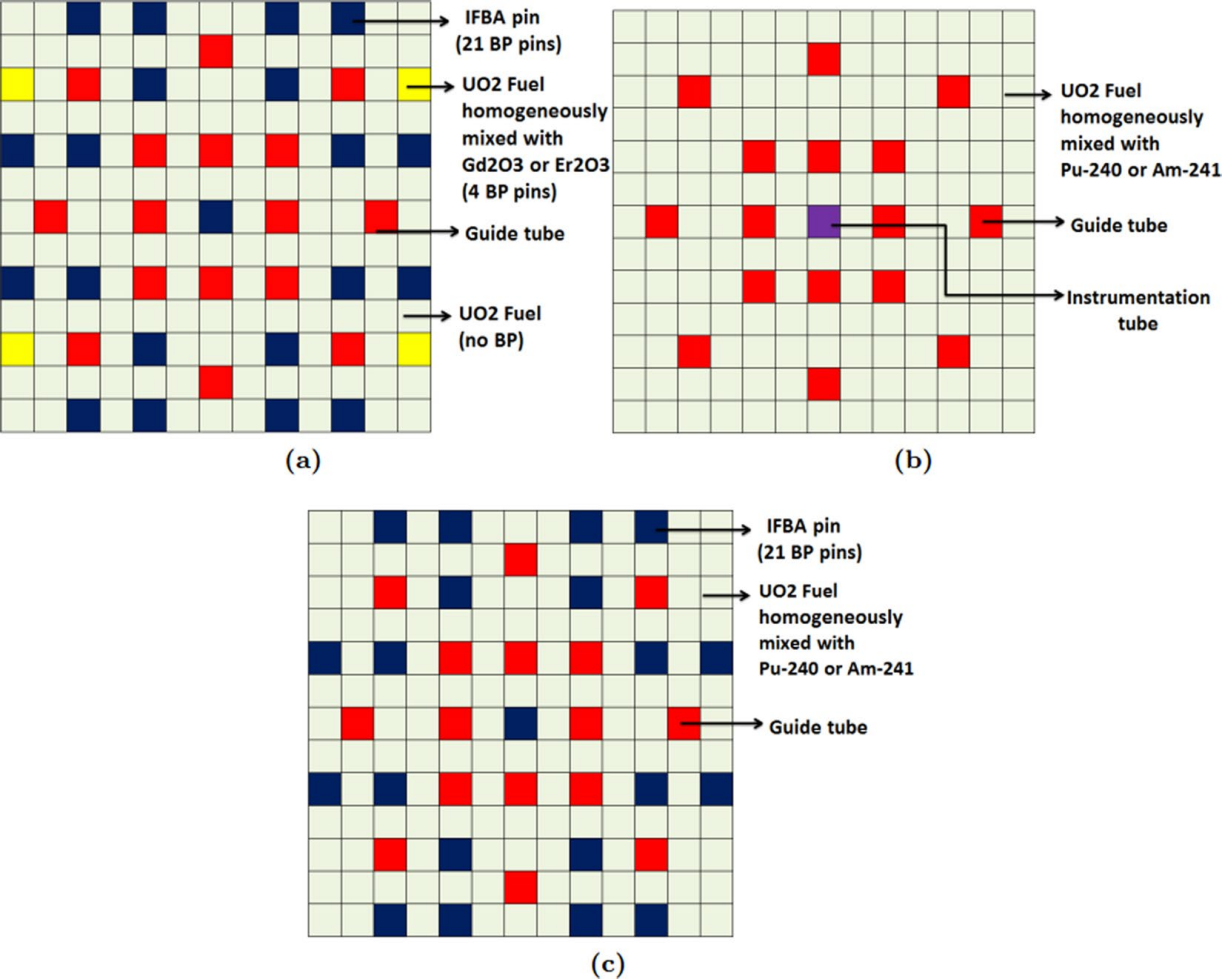
Different BP candidates: (**a**) Case 1: Composite BP: gadolinia-IFBA or erbia-IFBA; (**b**) Case 2: Pu-240 or Am-241 mixed homogeneously with UO_2_ fuel; (**c**) Composite BP: IFBA-Pu or IFBA-Am.

Finally, we investigate another composite BP: Pu-240 or Am-241 mixtures with high-thickness IFBA pins. Candidate BPs will be compared with the IFBA-only case for UO_2_ fuel. For the candidate BP cases, discharge burnup was calculated at ${k}_{\infty }=1$ (neglecting leakage). These cases are shown in Fig. 3.

It is important addressing that Pu-240 is commercially available (e.g. from Oak Ridge National Laboratory) in very small quantities proportionate with commercial demand. The material itself is produced readily in commercial reactor fuel from which it can be separated via well-established reprocessing techniques. Internationally, the separation of plutonium from spent nuclear fuel has been undertaken extensively in the production of mixed oxide (MOX) fuel. Moreover, separation of Am-241 produced from the decay of Pu-241 in spent fuel is undertaken commercially for industrial applications (radioisotope source for instrumentation, e.g. gauges and smoke detectors). Several kilograms of Am-241 are produced per year, a quantity that could be scaled up if the commercial need was significant. Concerning non-proliferation, weapons-grade plutonium is deemed to have a below threshold quantity of Pu-240 as, because it has a similar cross-section to Pu-239 yet is not fissile, it competes for neutrons and renders the Pu-239 useless for weapons manufacture. Pu-241 produced through the capture of neutrons in Pu-240 is fissile, having a threefold higher cross-section than for neutron capture, Pu-241 is very radioactive, decaying to Am-241, and is hence too radioactive for use in weapons production. Recall that the Pu-241 will exist in the presence of Pu-240 and Am-241, all of which compete for neutrons, Am-241 is fissile yet has a 30-fold higher n-gamma cross-section so is not a significant risk. Obviously, all of these materials are nuclear materials and handled accordingly. It is anticipated that these burnable poisons be integrated during fuel production, like for MOX.

## Investigation and Design of Improved Burnable Poisons

### Case 1: 21 IFBA pins with gadolinia/erbia

In this composite BP design, gadolinia (or erbia) and IFBA were used in the same lattice, however, in separate fuel rods. By precisely choosing the concentration and position of each BP, the assembly-level PPF is decreased. We have also examined the coexistence of Gadolinia/erbia and IFBA within the same fuel rod; however, the results were not promising. We observed that the external IFBA layer acted as a shielding by capturing neutrons. Therefore, depletion of gadolinia/erbia is delayed until the IFBA is fully consumed. This results in a greater residual burnup penalty, which necessarily shortens core life. Therefore, we have considered IFBA and gadolinia/erbia in separate fuel rods (Fig. 3(a)). Here we have considered high-thickness IFBA 150 μm pins and 10–30% Gd_2_O_3_ (Gd-IFBA) and Er_2_O_3_ (Er-IFBA).

In this composite BP design, gadolinia (or erbia) and IFBA were used in the same lattice, however, in separate fuel rods. By precisely choosing the concentration and position of each BP, the assembly-level PPF is decreased. We have also examined the coexistence of Gadolinia/erbia and IFBA within the same fuel rod; however, the results were not promising. We observed that the external IFBA layer acted as a shielding by capturing neutrons. Therefore, depletion of gadolinia/erbia is delayed until the IFBA is fully consumed. This results in a greater residual burnup penalty, which necessarily shortens core life. Therefore, we have considered IFBA and gadolinia/erbia in separate fuel rods (Fig. 3(a)). Here we have considered high-thickness IFBA 150 μm pins and 10–30% Gd_2_O_3_ (Gd-IFBA) and Er_2_O_3_ (Er-IFBA).

**Figure 4:**
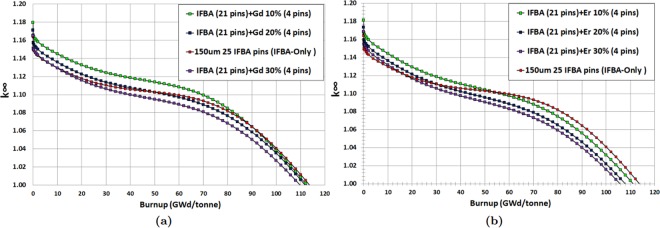
*k*_∞_ vs. burnup for Case 1 BP candidates: (**a**) gadolinia-IFBA and (**b**) erbia-IFBA.

It can be observed from Fig. 4(a,b) that Gd-IFBA and Er-IFBA are promising BP designs for controlling reactivity swing later in life (compared to the IFBA-only BP) if higher concentrations (>10%) of Gd_2_O_3_ and Er_2_O_3_ are employed. However, these designs cannot outperform the IFBA-only BP with regards to BOL reactivity suppression since the crucial self-shielding effect is reduced due to the decrease in the IFBA-only pins.

**Figure 5:**
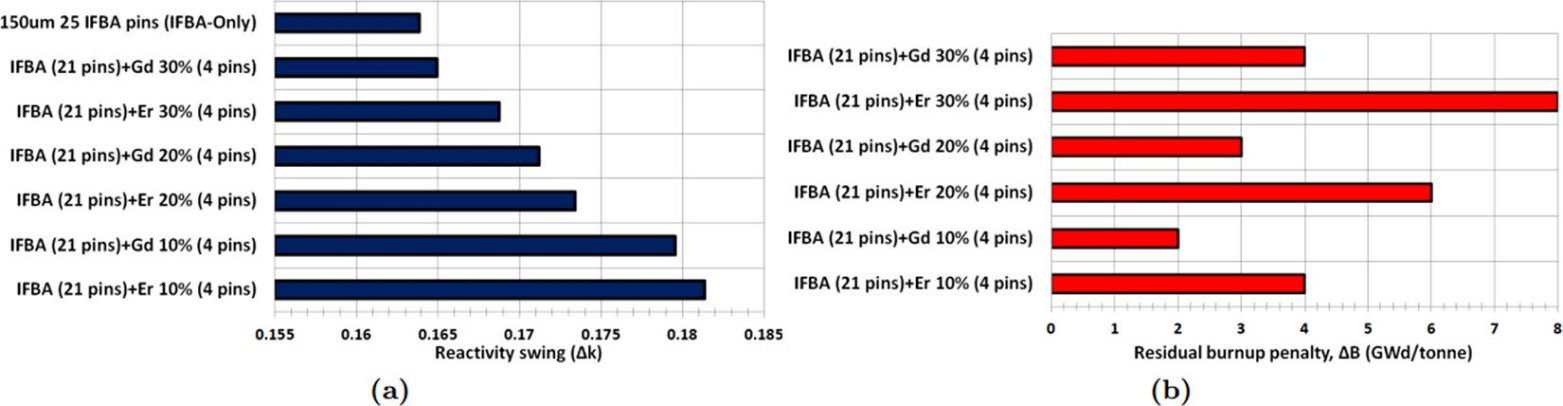
(**a**) Reactivity swing for Case 1 BPs; (**b**) Residual burnup penalty for Case 1 BPs.

The result shows that Gd-IFBA outperforms Er-IFBA concerning the performance of reactivity swing (Fig. 5(a)) and BOL reactivity suppression due to erbia’s much lower cross section compared to gadolinia. For this reason, erbia does not provide the same strong self-shielding^41^ and, therefore, suffers from larger EOL residual burnup penalties compared to gadolinia (Fig. 5(b)), which inevitably shortens the discharge burnup and core life. We observed that if we increase Gd_2_O_3_ or Er_2_O_3_ concentration, we obtain a longer cycle at the expense of reduced reactivity suppression. IFBA-only design is used as a reference, and in order to achieve the poisoning equivalent of the reference, Gd_2_O_3_ should be used. If using erbium is desired, then the poisoning mode must be altered because it has a low absorption cross section. In addition, the erbium has to be highly diluted with a large number of rods. This results in a significant modification of the neutron 17 as a whole.

We conclude from our investigation of this BP strategy that use of the Gd-IFBA design can lead to a ~16% IFBA pins reduction while exhibiting the similar BP performance as for the IFBA-only design.

### Case 2: Homogeneously mixed Pu-240 or Am-241 with UO_2_ fuel

**Figure 6:**
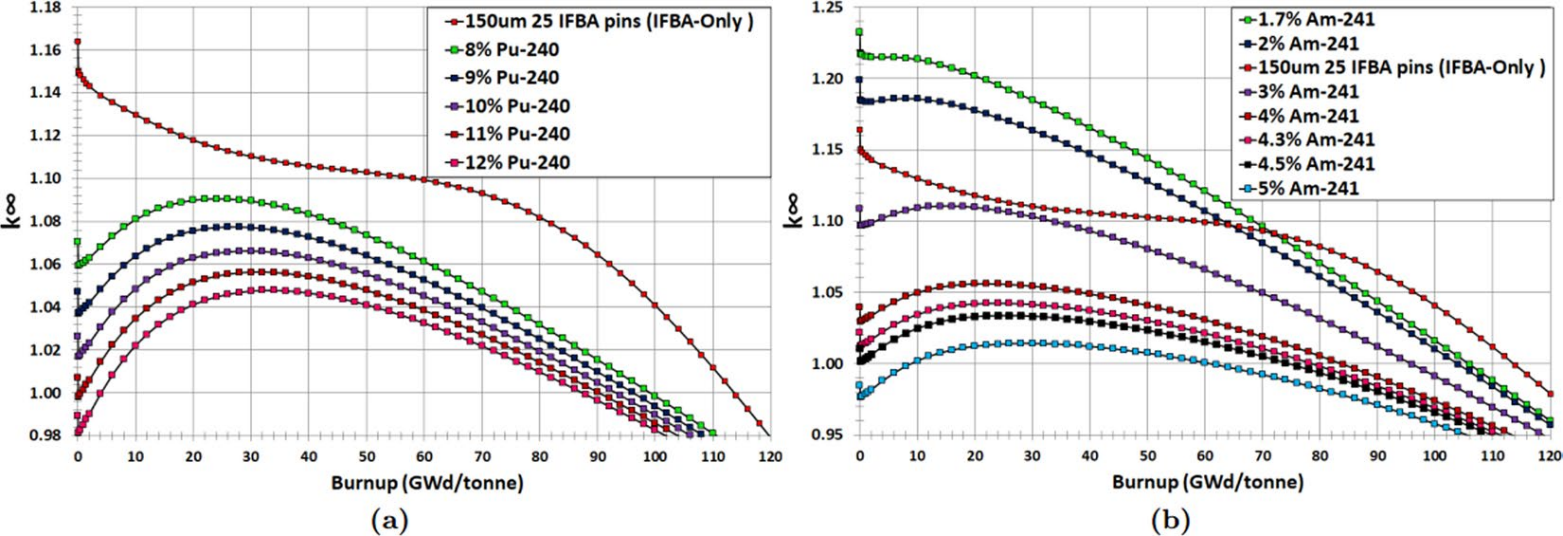
*k*_∞_ vs. burnup for for Case 2 BP candidates: (**a**) homogeneously mixed Pu-240 with UO_2_ fuel; (**b**) homogeneously mixed Am-241 with UO_2_ fuel.

This study also pursues the novel idea of mixing Pu-240 or Am-241 homogeneously into the fuel^39^. Pu-240 is selected because of its high resonance absorption (~10^5^ barns) in the vicinity of ~1.04 eV in comparison with Pu-238 (~10^3^ barns at ~2.85 eV), as shown in Fig. 2. This results in Pu-240 being more efficient in suppressing excess reactivity. Figure 6(a,b) show that the homogeneous admixture of Pu-240 or Am-241 into the fuel substantially suppress BOL reactivity due to their much larger thermal capture cross sections than that of U-238 (1.73 barns). This allows the number of BP rods used for through-life reactivity swing and excess reactivity control to be reduced. Since the homogeneous mixture of Pu-240 or Am-241 homogeneously into the fuel regulates the self-shielding effect, BOL reactivity suppression is almost linearly proportional to the amount of Pu-240 or Am-241.

Homogeneous mixing of Pu-240 with UO_2_ gives promising results compared to the IFBA-only design. It can be seen from Fig. 6(a) that homogeneous mixing of 8% Pu-240 reduces the reactivity swing by ~55% and exhibits ~9% more BOL reactivity suppression compared to the IFBA-only design while achieving a discharge burnup of ~100 GWd/tonne. A drawback is that it provides ~14 GWd/tonne reactivity penalty, which has a detrimental effect on core life. This suggests that reducing the amount of Pu-240 would lead to a smaller burnup penalty at the cost of less reactivity suppression and more swing.

Am-241 mixed homogeneously with the fuel exhibits the same characteristics for the same reasons, although there are differences in the amounts of initial excess reactivity suppression, reactivity swing and burnup penalty. It can be seen from Fig. 6(b) that 3% Am-241 mixed with the fuel contributes to ~30% less reactivity swing and ~3% more suppression compared to the IFBA-only design while achieving a discharge burnup of ~100 GWd/tonne, which clearly identifies a potential BP saving. An admixture of even relatively small amounts of Am-241 (e.g. 3% Am-241 concentration) strongly influences the local thermal flux and results in a marked depression of the local ${k}_{\infty }$, which ultimately influences the overall ${k}_{\infty }$ of the entire subassembly.

**Figure 7:**
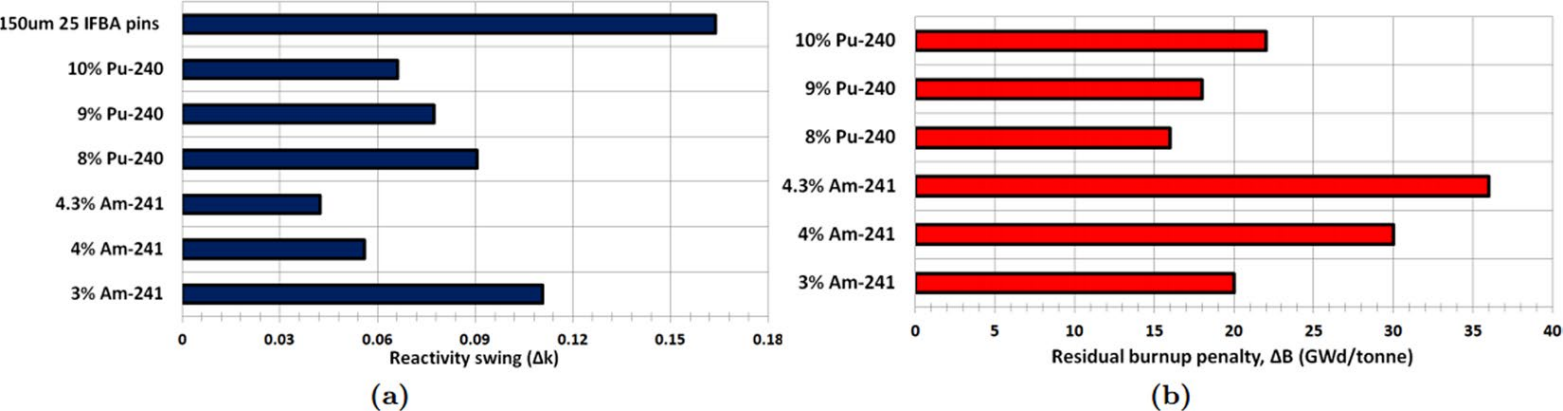
(**a**) Reactivity swing for Case 2 BPs; (**b**) Residual burnup penalty for Case 2 BPs.

It is clear that Am-241 exhibits better BP performance than that of Pu-240 concerning the BOL reactivity suppression and reactivity swing (Fig. 7(a)), although Am-241 suffers from a larger EOL residual burnup penalty (Fig. 7(b)). This is due to the very slow burn-out of the Am-241 mixed fuel, which leads to a significant residual absorption penalty. Utilizing higher initial enrichment might be a solution to avoid this problem. The amount of reactivity suppressed at BOL increases with the addition of Pu-240 or Am-241 compared to the IFBA-only design. Therefore, the BP requirements can be reduced by using Pu-240 or Am-241 mixed homogeneously with the fuel.

### Case 3: 21 IFBA pins with homogeneously mixed Pu-240 or Am-241 with UO_2_ fuel

**Figure 8:**
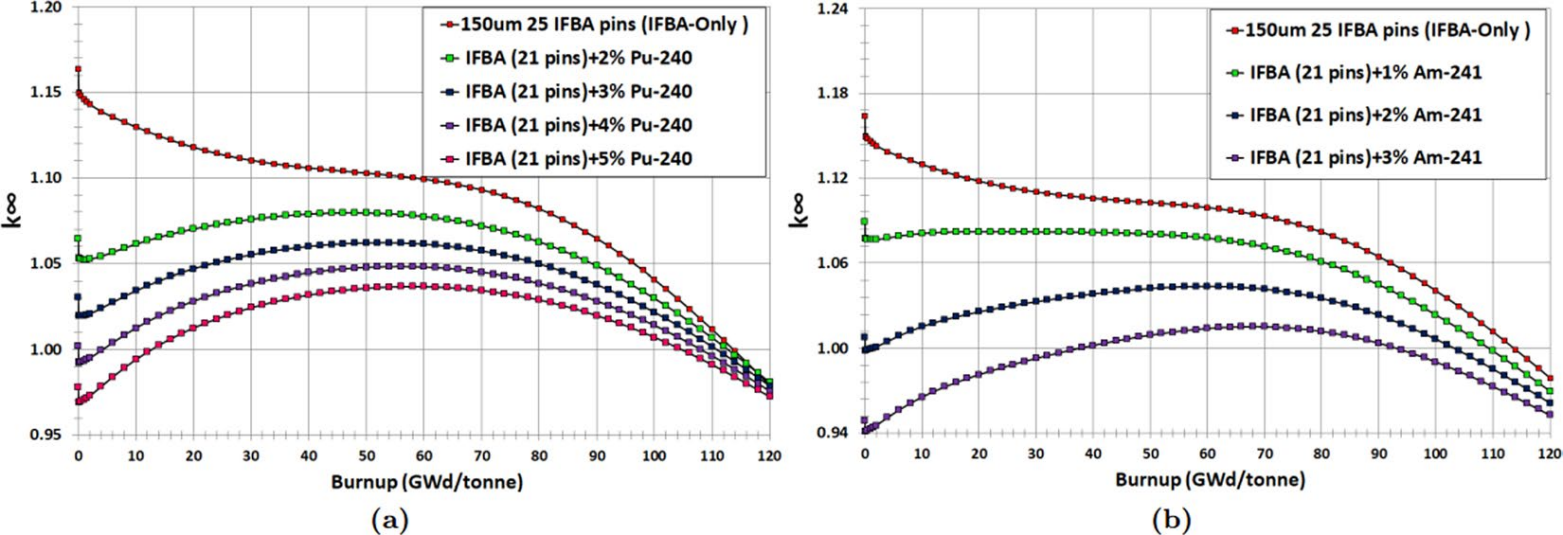
*k*_∞_ vs. burnup for Case 3 BP candidates: (**a**) IFBA-Pu; (**b**) IFBA-Am.

Finally, we have observed promising results for the IFBA-Pu-240 (IFBA-Pu) and IFBA-Am-241 (IFBA-Am) configurations (Case 3). In this strategy, 21 IFBA pins were used and Pu-240 or Am-241 were mixed homogeneously with the fuel. It can be seen from Fig. 8(a) that the IFBA-Pu configuration with 3% Pu-240 results in a ~60% lower reactivity swing and ~8% more reactivity suppression compared to the IFBA-only design while providing almost the same cycle length. Another promising configuration is the IFBA-Am mixture (Fig. 8(b)) with 2% Am-241, which offers better reactivity swing and suppression performance but with ~2 GWd/tonne less cycle length than the IFBA-Pu configuration.

**Figure 9:**
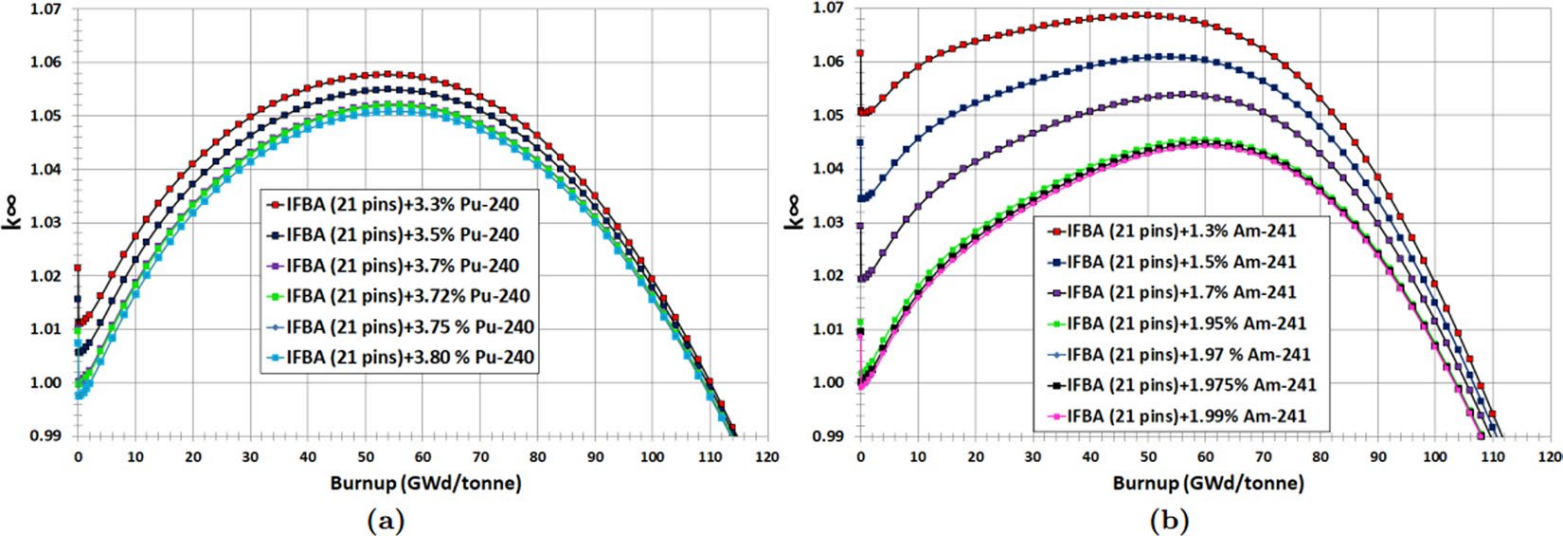
*k*_∞_ vs. burnup for Case 3 BP candidates: (**a**) Optimization of Pu-240 content of composite BP IFBA-Pu; (**b**) Optimization of Am-241 content of composite BP IFBA-Am.

We can conclude from this study that composite BPs such as the IFBA-Pu and IFBA-Am designs are better BP designs than the IFBA-only design in terms of reactivity swing and suppression while providing comparable cycle lengths. However, these Case 3 BP designs require further optimization in order to achieve the minimum possible BOL ${k}_{\infty }$ and reactivity swing while maintaining a discharge burnup of ≥100 GWd/tonne. Figure 9(a,b) show that IFBA-Pu with 3.70% Pu-240 and IFBA-Am with 1.975% Am-241 can provide ~70% reduced swing and ~10% more reactivity suppression compared to the IFBA-only design while yielding cycle lengths of ≥100 GWd/tonne.

**Figure 10:**
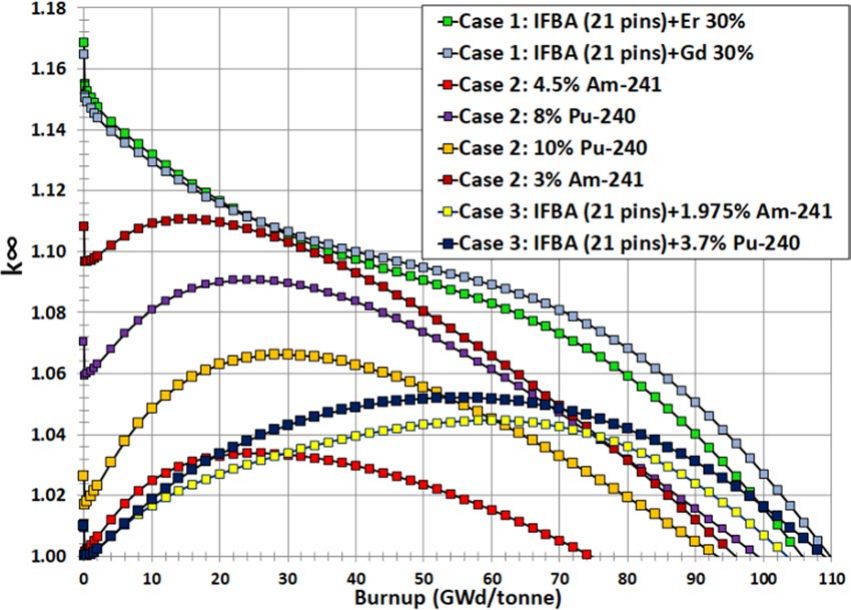
Comparison of different BP candidate design cases.

IFBA-Pu and IFBA-Am designs exhibit better BP performance compared to the previously considered cases (Cases 1 and 2). This is due to the fact that an IFBA 150 μm absorbs ~95% of incident neutrons since this poison layer consists of a thickness of 3*λ*, where *λ* is the neutron mean free path. This leads to the severely attenuated neutron flux in fuel pin early in life. However, later in life, fuel pin gradually experiences power as the BP burns off. As expected, late in life, higher fissile content will be present in the BP-coated fuel pins, allowing the assembly to stay critical even longer than if it had no poison at all. With this (Case 3) BP configuration, minimum reactivity swing and higher BOL reactivity suppression compared to Cases 1 and 2 can be obtained while achieving a discharge burnup of ~100 GWd/tonne (Fig. 10).

This design study suggests that the Case 3 (IFBA-Pu and IFBA-Am) designs are to be preferred for our SBF marine core application. BP designs using 21 IFBA pins with homogeneously mixed 3.70% Pu-240 (IFBA-Pu) or 1.975% Am-241 (IFBA-Am) will therefore be used in our whole-core analyses.

## Power Peaking Factors

**Figure 11:**
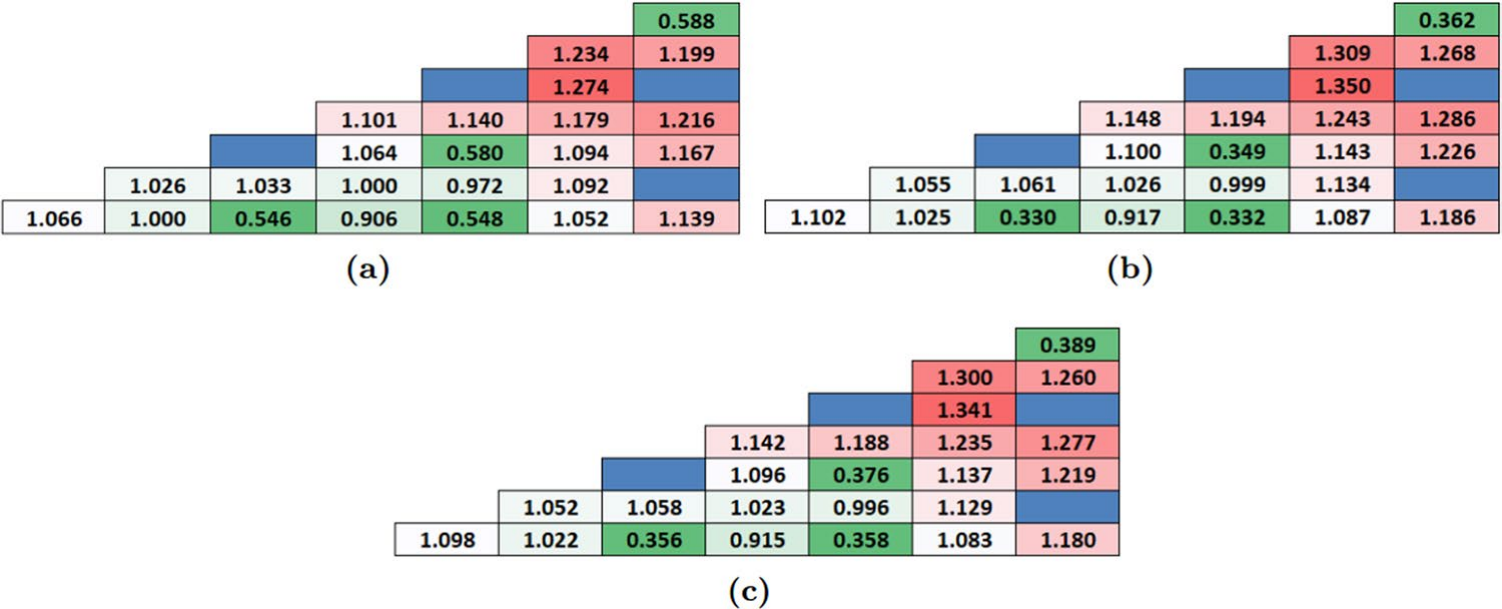
BOL pin power distribution of one octant assembly: (**a**) 150 μm 25 IFBA pins; (**b**) Gadolinia-IFBA (30% gadolinia with 4 pins–IFBA with 21 pins); (**c**) Erbia-IFBA (30% erbia with 4 pins–IFBA with 21 pins).

BOL subassembly-level PPFs for one octant assemblies have been considered for Cases 1–3. It can be observed for the Case 1 alternative BPs that UO_2_ assemblies exhibit PPF of 1.35 and 1.34 with 30% gadolinia-IFBA (Fig. 11(b)) and 30% erbia-IFBA (Fig. 11(c)) respectively, which are ~6% higher than the IFBA-only BP with 25 absorber pins (PPF of 1.28).

**Figure 12:**
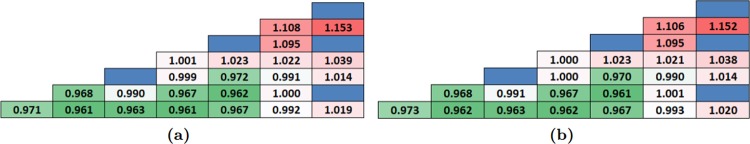
BOL pin power distribution of one octant assembly for homogeneously mixed actinides with UO_2_ fuel: (**a**) 8% ^240^Pu; (**b**) 3% ^241^Am.

Furthermore, Case 2 alternative BPs exhibit excellent improvement in PPF values of 1.153 and 1.152 with 8% ^240^Pu (Fig. 12(a)) and 3% ^241^Am (Fig. 12(b)) respectively, which are ~10% lower than the IFBA-only BP with 25 absorber pins. Case 2 alternative BPs show improvements in controlling PPF, although these exhibit significant discharge burnup penalty.

**Figure 13:**
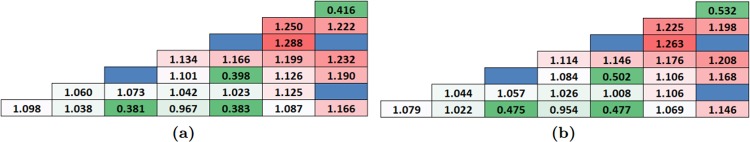
BOL pin power distribution of one octant assembly: (**a**) IFBA-Pu (3.70% Pu-240); (**b**) IFBA-Am (1.975% Am-241).

In addition, It can also be observed in Fig. 13 for Case 3 that UO_2_ assemblies exhibit PPF of 1.29 and 1.26 with IFBA-Pu (3.70% Pu-240) and IFBA-Am (1.975% Am-241), respectively. It is important noting that addressing that non-IFBA options (Case 2) exhibit the lowest PPF, while IFBA-Pu and IFBA-Am (Case 3) exhibit comparable PPF values to IFBA-only case.

## Whole-Core Feasibility

In Section 4.3, we developed a subassembly design with BPs that was optimized for controlling reactivity swing for obtaining target core lifetime of 15 years. In the previous phase of the assembly-level BP studies, WIMS performs deterministic neutron transport calculations for every pin in a fuel assembly; however, it does these calculations for an infinitely large core, neglecting the finite height and radius of an actual reactor. Hence, WIMS cannot describe the effects of neutron leakage. To explore these essential aspects of whole-core reactor design, we use the whole-core code PANTHER by loading these assemblies. The main objectives of this whole-core feasibility analyses are to confirm the criteria below while reducing 20% IFBA use:

1. To confirm that the subassembly design with the alternative BP has sufficient reactivity for the desired 15-year operation period.
2. To ensure the lowest possible through-life reactivity swing (below 4000 pcm) in order to reduce the dependency on control rods.
3. To maintain whole-core radial form factor (RFF) below the industrial limit of 1.50 over life to prevent spatial power-peaking.
4. To evaluate the key neutronic safety parameters to confirm that the reactivity feedback (fuel and moderator temperature coefficient) are negative and axial offset (AO) values are within the acceptable range from −10% to +10%.

**Figure 14:**
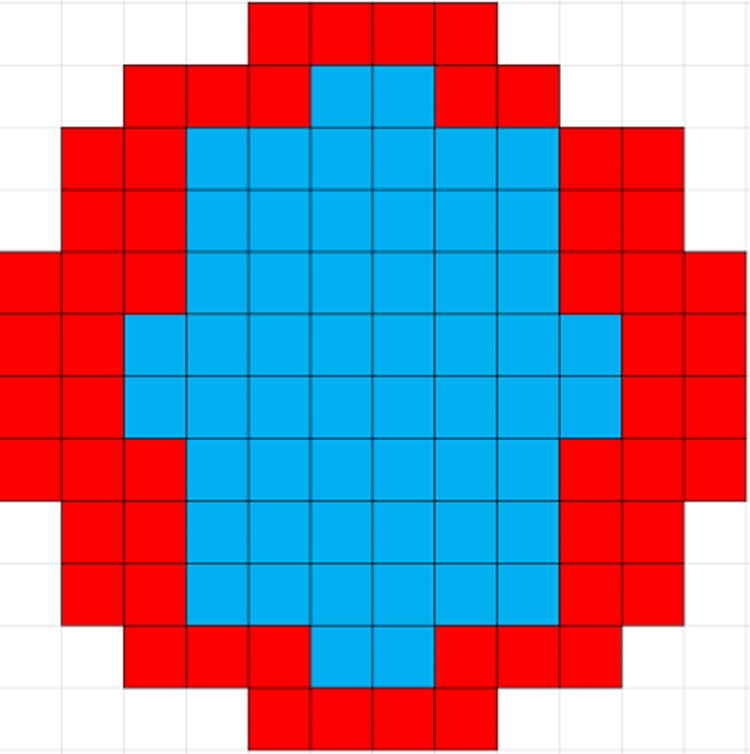
Radial zoning whole-core loading pattern in the 112-assembly SMR core: blue = A (56 assemblies), red = B (56 assemblies). Blue denotes IFBA-Pu or IFBA-Am assemblies and red denotes IFBA-only assemblies.

This whole-core study models the 112-subassembly core^10^ using PANTHER and divide it into two radial zones (A and B, see Fig. 14) while considering the hot full power operating conditions (fuel temperature = 900 K, coolant temperature = 580 K and coolant density = 707 kg/m^3^) and all control rods out conditions. We consider a radial-zoning loading pattern (LP) scheme in which assemblies in Zones A and B have IFBA-Pu or IFBA-Am and IFBA-only assemblies, respectively. Since we observed in Sect.4.3 that Case 3: 3.70% Pu-240 (IFBA-Pu) or 1.975% Am-241 (IFBA-Am) assemblies exhibit a burnup penalty compared to the IFBA-only assembly, 50% (56) of the locations are loaded with IFBA-only assemblies in order not to compromise the desired core lifetime of 15 years. Comparisons are made with the full core loaded entirely with IFBA-only assemblies.

**Figure 15:**
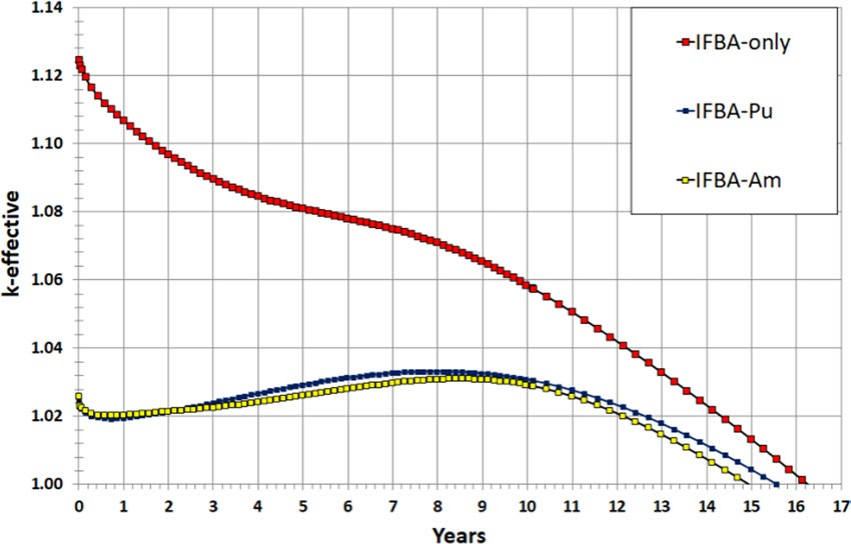
*k*_eff_ vs. time (years) for the radial-zoning core LP.

**Figure 16:**
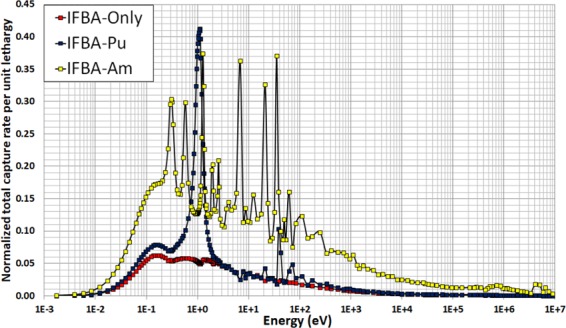
BOL Normalized total neutron capture rates per unit lethargy for the candidate cores.

It can be seen in Fig. 15 that both the IFBA-Pu and IFBA-Am cores are successful in achieving the target core lifetime of 15 years for the proposed SBF SMR core. The IFBA-Pu core can achieve a ~6% (272 days) longer core life than the IFBA-Am core. Both the IFBA-Pu and IFBA-Am cores exhibit a shorter core lifetime than the IFBA-only core due to the residual burnup penalty associated with their corresponding BP designs. On the other hand, the IFBA-Pu and IFBA-Am cores exhibit ~10% higher BOL reactivity suppression and ~70% lower reactivity swings (below the targeted 4000 pcm) than the IFBA-only core while reducing 20% IFBA use, the former due to their much higher capture rates per unit lethargy over the thermal energy range below 1 eV (Fig. 16).

**Figure 17:**
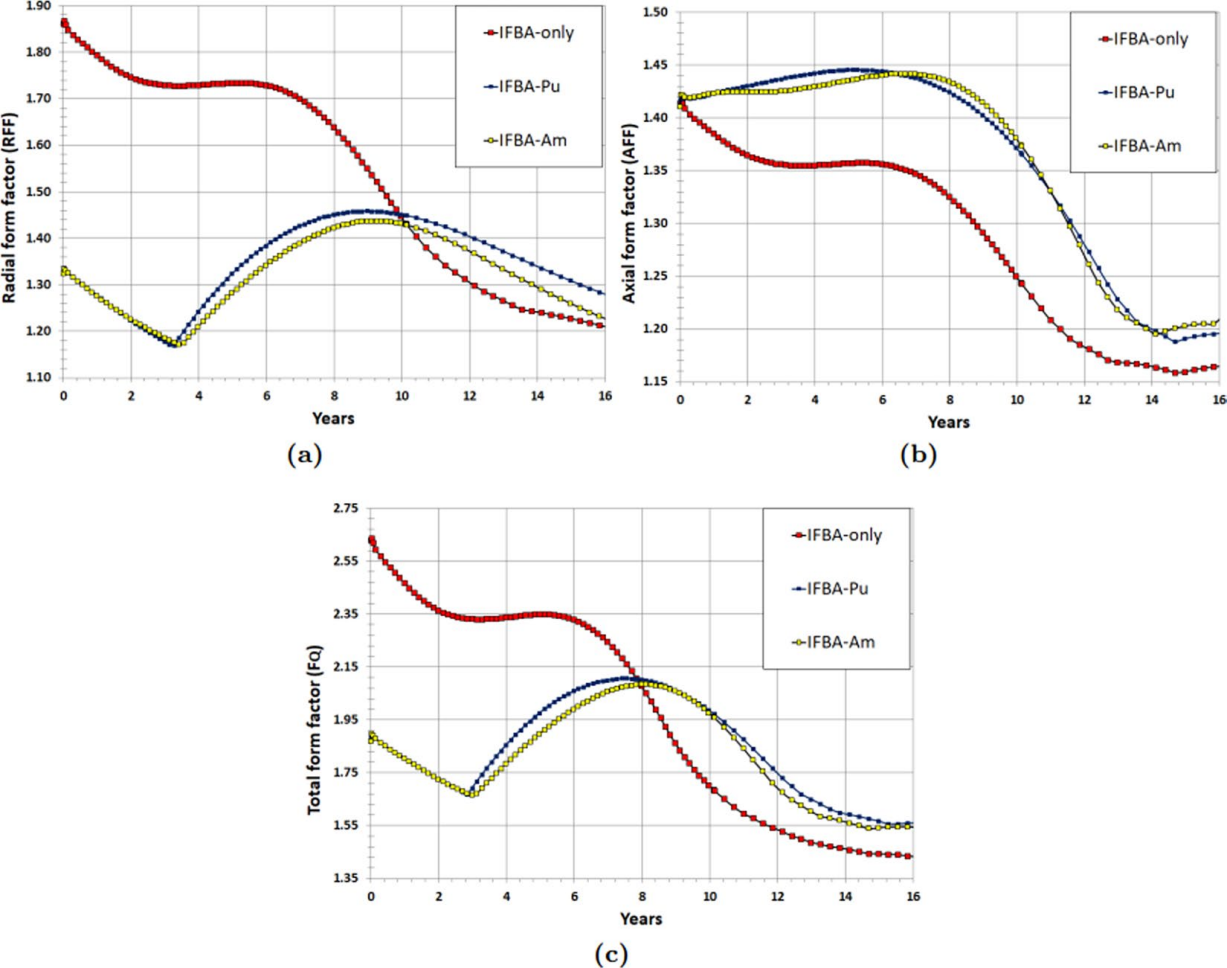
Form factors as a function of time (years) for the radial-zoning core LP: (**a**) RFF; (**b**) AFF; (**c**) *F*_*Q*_.

Figure 17(a) shows the variation of the radial form factor (RFF) over the core lifetime for the SMR cores. IFBA-only core experiences a maximum RFF of ~1.9 at the BOL due to the very high power share in inner subassemblies^10^. In contrast, IFBA-Pu and/or IFBA-Am can maintain the through-life RFF values below 1.50. Figure 17(b,c) show the variation of the axial form factor (AFF) and total form factor (*F*_*Q*_), respectively. The values of AFF and *F*_*Q*_ should not exceed 1.60 and 2.52, respectively, at hot full power^10,39^. It can be seen that both the IFBA-Pu and IFBA-Am cores can maintain the through-life AFF and *F*_*Q*_ values below their allowable limits, while the IFBA-only core exceeds these limits. It is worthwhile addressing that IFBA-Pu and IFBA-Am cores can achieve ~30% lower RFF and ~28% lower *F*_*Q*_ compared to the IFBA-only core.

**Figure 18:**
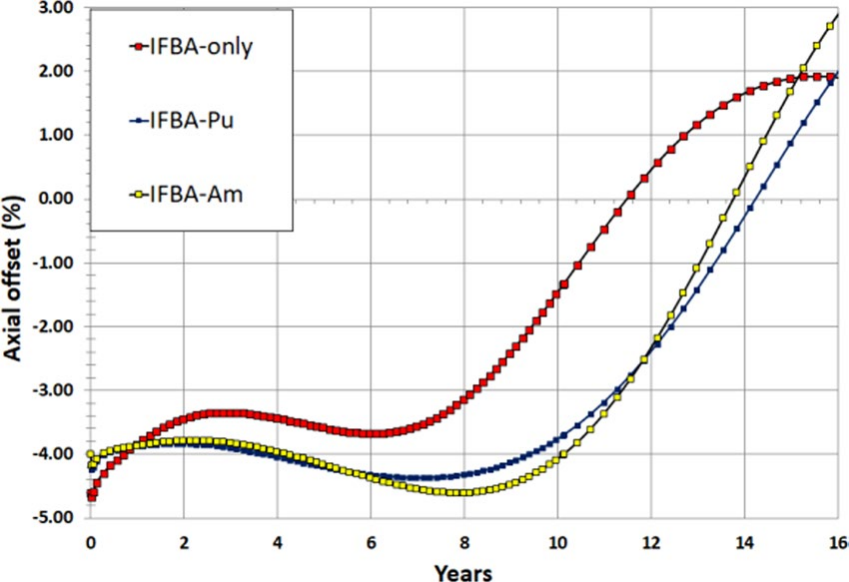
Axial offset (%) as a function of time (years) for the radial-zoning core LP.

The axial offset (AO) is the ratio of the normalized power difference between the top and bottom halves of the core. This is a measure of the axial imbalance of power generation and is sought to be minimised to control the AFF. The recommended AO values should be in the range of −10% to +10%^10,39^. Figure 18 indicates that IFBA-only, IFBA-Pu and IFBA-Am perform reasonably favourably according to this measure while experiencing the through-life AO values from ~−4.5% to ~+3% but this is the result of a complex coupling of neutronics and thermal-hydraulic feedback. It is also observed that due to the higher AFF experienced by the IFBA-Pu core early in life, its AO is slightly higher than that of the IFBA-Am core for the first 6 years. Later in life, the IFBA-Am core exhibits a higher AO due to its higher AFF. Hence, it is concluded that this AO characteristic is coherent with the AFF values. The higher burnup in the lower portion of the fuel assembly later in life, leading to the depletion of fissile nuclei in this region, is the cause of the swing toward a positive AO for all the cores. However, average AO values are negative due to the efficient moderation and higher fissile concentration in the bottom of the cores^10^.

**Figure 19:**
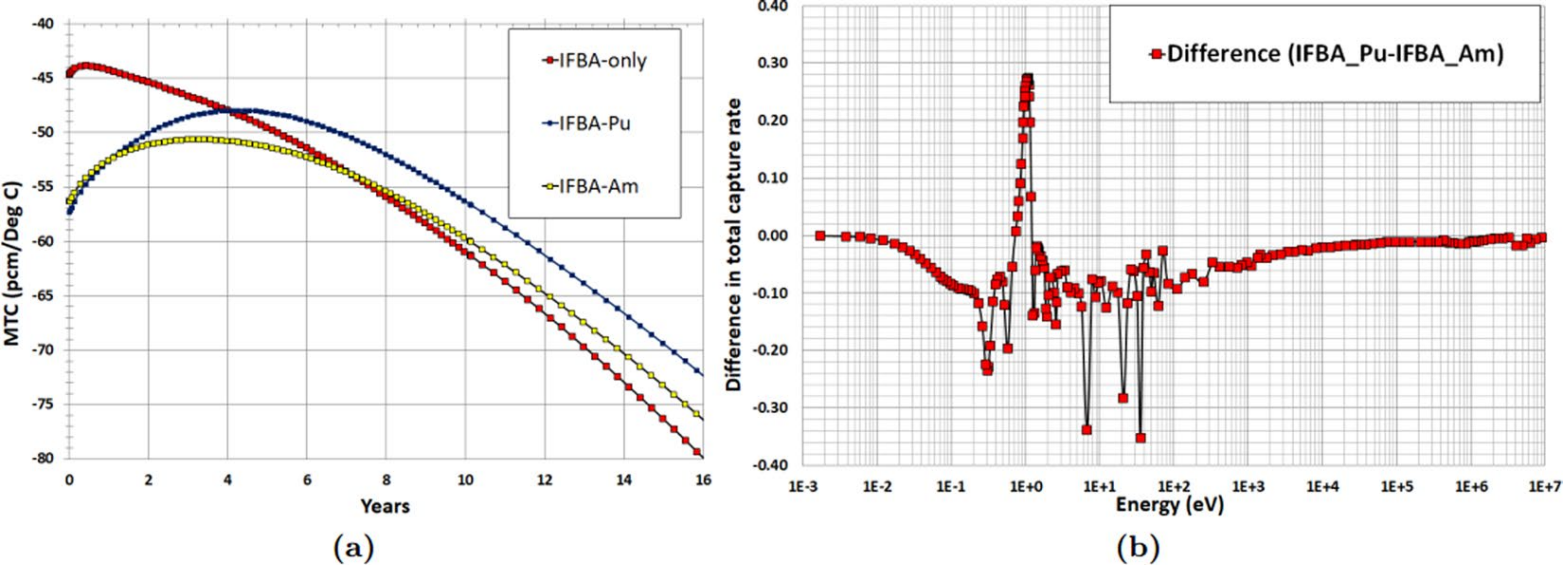
(**a**) MTC as a function of time (years) for the radial-zoning core LP; (**b**) Difference in total BOL neutron capture rate between IFBA-Pu and IFBA-Am.

Two important reactivity feedback parameters: moderator temperature coefficient (MTC) and fuel temperature coefficient (FTC) are evaluated in the whole-core feasibility study. The MTC is an indication of the intrinsic stability of the reactor to coolant temperature, and therefore power, transients. The more negative this parameter is, the more quickly the reactor will tend to stabilise itself if the coolant temperature rises due to a peak in power. Figure 19(a) illustrates the strong negative MTC over core life since no soluble boron is used in the proposed SMR core. IFBA-Pu core exhibits marginally more negative MTC value than the IFBA-Am core at BOL due to the large resonances in the vicinity of ~1.1 eV (Fig. 16)^10,41^. Figure 19(b) shows the difference in total neutron capture rate between that IFBA-Pu and that IFBA-Am core. It can be observed that IFBA-Pu core exhibits a higher total neutron capture rate than that of the IFBA-Am core in the vicinity of ~1.1 eV^10^.

**Figure 20:**
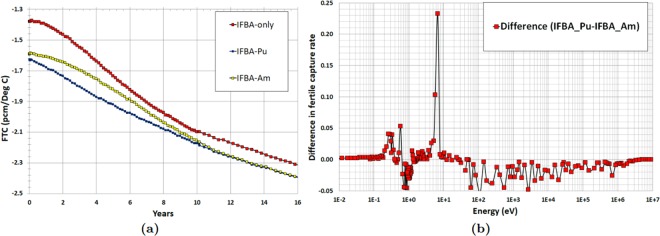
(**a**) As a function of time (years) for the radial-zoning core LP; (**b**) Difference in fertile capture rate between IFBA-Pu and IFBA-Am.

Figure 20(a) confirms that FTC values are negative throughout the core lifetime for the candidate SMR cores and IFBA-Pu core shows considerably more negative FTC values than that of the IFBA-Am core. Figure 20(b) shows the difference in fertile capture rate between that IFBA-Pu and that IFBA-Am core. It can be observed that IFBA-Pu core exhibits higher fertile capture rate than that of the IFBA-Am core in the vicinity of ~6.8 eV^10^.

## Conclusions

This study focused on the design and neutronic performances of alternative BP loading schemes to reduce the use of 150 μm IFBA in the proposed soluble-boron-free SMR core since B-10 in IFBA undergoes an (n, α) capture reaction, and the resulting helium raises the pressure within the plenum and in the cladding. This paper considered three novel types and strategies of BP loading scheme: (Case 1) a composite BP: gadolinia (Gd_2_O_3_) or erbia (Er_2_O_3_) with 150 μm thickness ZrB_2_ IFBA; (Case 2) Pu-240 or Am-241 mixed homogeneously with the fuel; (Case 3) another composite BP: Pu-240 or Am-241 with 150 μm thickness ZrB_2_ IFBA. All the BP candidates were compared with the reference high-thickness IFBA-only (150 μm 25 IFBA pins) BP design for UO_2_ fuel. The key findings of this neutronic study are:

1. Case 1 (21 IFBA Pins with 4 gadolinia/erbia pins): Gd-IFBA outperforms Er-IFBA concerning the performance of reactivity swing and BOL reactivity suppression. The use of the Gd-IFBA design can lead to a ~16% IFBA pins reduction while exhibiting the similar BP performance as for the IFBA-only design.
2. Case 2 (Homogeneously mixed Am-241/Pu-240): Am-241 exhibits better BP performance than that of Pu-240 concerning the BOL reactivity suppression and reactivity swing, although Am-241 suffers from a larger EOL residual burnup penalty.
3. Case 3 (21 IFBA pins with homogeneously mixed Pu-240 or Am-241): IFBA-Pu (3.70% Pu-240) and IFBA-Am (1.975% Am-241) designs are better BP designs than the IFBA-only design in terms of reactivity swing and suppression while providing comparable cycle lengths. IFBA-Pu with 3.70% Pu-240 and IFBA-Am with 1.975% Am-241 can provide ~70% reduced swing and ~10% more reactivity suppression compared to the IFBA-only design while yielding cycle lengths of ≥100 GWd/tonne. This design study suggested that the Case 3 (IFBA-Pu and IFBA-Am) designs are to be preferred for our SBF marine core application.
4. Non-IFBA options exhibit the lowest PPF, while IFBA-Pu and IFBA-Am (Case 3) exhibit comparable PPF values to IFBA-only case.
5. In the whole-core study, Case 3 BPs (IFBA-Pu and IFBA-Am cores) are successful in achieving the target core lifetime of 15 years for the proposed SBF SMR core. The IFBA-Pu core can achieve a ~6% (272 days) longer core life than the IFBA-Am core. Case 3 BPs contributes to ~10% higher BOL reactivity suppression, ~70% lower reactivity swings ~30% lower radial form factor and ~28% lower total peaking factor compared to the IFBA-only core while reducing 20% IFBA use for reactivity control.

It can be concluded from this design study that the proposed composite BPs using Pu-240 and Am-241 with IFBA (IFBA-Pu and IFBA-Am) are the preferred burnable poison option for a SBF and long life SMR marine core since these admixtures result in larger initial excess reactivity suppression, reduced reactivity swing, lower power peaking factors and, thus, higher potential BP savings compared to the IFBA-only design while reducing 20% IFBA use. The higher BOL reactivity suppression and smaller reactivity swing of IFBA-Pu and IFBA-Am make the task of reactivity control in a long life SBF core easier than that of the traditional burnable poisons. These composite BPs exhibit a small residual burnup penalty at EOL compared to the IFBA-only design.

The scope of this paper is only limited to the neutronic feasibility of candidate BPs. Future work will also consider the neutronic and practical implications (availability of the fuel, problems of separation and manufacture) and proliferation concerns of these proposed BPs.
